# A survey of pharmacists’ perception of foundation level competencies in African countries

**DOI:** 10.1186/s12960-018-0280-1

**Published:** 2018-04-02

**Authors:** Arit Udoh, Andreia Bruno, Ian Bates

**Affiliations:** 1 University College London School of Pharmacy, London, United Kingdom; 2 Faculty of Pharmacy and Pharmaceutical Sciences, Monash University (Parkville Campus), Melbourne, Australia

**Keywords:** Competencies, Professional development, Pharmacy practice, Pharmacy workforce

## Abstract

**Background:**

Evidence from published literature in pharmacy practice research demonstrate that the use of competency frameworks alongside standards of practice facilitate improvement in professional performance and aid expertise development. The aim of this study was to evaluate pharmacists’ perception of relevance to practice of the competencies and behaviours contained in the FIP Global Competency Framework (GbCF v1). The overall objective of the study was to assess the validity of the GbCF v1 framework in selected countries in Africa.

**Methods:**

A cross-sectional survey of pharmacists practicing in 14 countries in Africa was conducted between November 2012 and December 2014. A combination of purposive and snowball sampling method was used. Data was analysed using SPSS v22.

**Results:**

A total of 469 pharmacists completed the survey questionnaire. The majority (91%) of the respondents were from four countries: Ghana, Kenya, Nigeria and South Africa. The study results showed broad agreement on relevance to practice for 90% of the behaviours contained in the GbCF v1 framework. Observed disagreement was associated with area of pharmacy practice and the corresponding patient facing involvement (*p* ≤ 0.05). In general, the competencies within the ‘pharmaceutical care’ and ‘pharmaceutical public health’ clusters received higher weighting on relevance compared to the research-related competencies which had the lowest. Specific inter-country variability on weighting of relevance was observed in five behaviours in the framework although, this was due to disparity in ‘degree of relevance’ that was related to sample composition in the respective countries.

**Conclusion:**

The competencies contained in the GbCF v1 are relevant to pharmacy practice in the study population; however, there are some emergent differences between the African countries surveyed. Overall, the findings provide preliminary evidence that was previously lacking on the relevance of the GbCF v1 competencies to pharmacy practice in the countries surveyed.

**Electronic supplementary material:**

The online version of this article (10.1186/s12960-018-0280-1) contains supplementary material, which is available to authorized users.

## Background

Competent pharmacists improve therapeutic outcomes, minimise the risk associated with medicines use and assure patient safety through the provision of medicine expertise [1–5]. The central role pharmacists play within the health system underpins the demand for a competent and highly skilled workforce that is equipped with the requisite knowledge and skills relevant to population health needs [6–8]. This is of particular importance in resource-limited settings such as in sub-Saharan Africa where severe workforce shortages hamper access to health services including medicines expertise [9, 10].

The International Pharmaceutical Federation (FIP) is the global leadership body representing 3 million pharmacists and pharmaceutical scientists worldwide [11]. FIP*Ed*, which is the pharmacy education and workforce development arm of the FIP, advocates the need to define and articulate the competencies that pharmacists require to consistently perform safely, effectively, and efficiently [12]. The overall objective is to provide an infrastructure for global guidance on the practice-based expectations of the pharmacy workforce [13].

Published research demonstrate that when competency frameworks are used alongside standards of practice: it facilitates improvement of pharmacists’ performance [14–17], promotes the attainment and maintenance of fitness to practice [17, 18], aids identification of knowledge gaps and learning needs [19], and fosters continuing professional development [20]. The findings of longitudinal studies show that using competency frameworks to identify knowledge gaps and tailor learning activities significantly improve pharmacists’ performance (*p* ≤ 0.05) at 6 months [14, 15, 17], 9 months [16], and 12 months [18, 20]. Similar findings from comparative studies conducted in United Kingdom [14, 20] indicate that competency frameworks facilitate a more sustained improvement in pharmacists’ performance (*p* < 0.001) in the intervention group at 12 months when compared to a control group that had no access to a framework. These findings have also been corroborated by other studies in Australia [15], Croatia [18], Serbia [17], and Singapore [16].

In 2012, FIP*Ed* developed the FIP Global Competency Framework (GbCF v1) using evidence-based methodologies [21]. This framework was specifically designed to serve as a source document containing the core competencies expected of foundation level pharmacy practitioners (this means, pharmacists with less than 5 years practice experience) [13]. Since its development, the GbCF v1 has been successfully used to design pre-service education and training curriculum in a number of countries [22, 23]. It has also been used to develop country-specific frameworks for in-service practitioners in Ireland [24], the Pacific Island countries [25], Croatia, Singapore and Serbia [21]. A previous survey that validated the GbCF v1 using evidence from 64 countries [26] showed that 70% of respondents ranked all the behaviours in the questionnaire as relevant to practice. However, respondents from countries in Africa comprised only 12.3% of the total sample in the survey [26]. The aim of this present study was therefore to evaluate pharmacists’ perception of the relevance to practice of the competencies and behaviours contained in the GbCF v1, focusing on selected African countries.

## Methods

### Data collection and sampling

A cross-sectional survey of pharmacists practicing in selected countries in Africa was conducted between November 2012 and December 2014. A combination of purposive and snow-ball sampling method was used. Due to a lack of access to the pharmacy membership list of the respective countries in Africa, the URL link to the online survey was circulated centrally via email to the 35 FIP member organisations in Africa for onward distribution to their respective individual members. The FIP member organisations contacted were the leadership bodies representing practicing pharmacists in 24 countries in Africa (list provided in the Appendix). These organisations were selected based on availability of contact persons, expression of interest to participate when contacted and willingness to gather data. The survey invite was also disseminated through the FIP United Nations Education Scientific and Cultural Organisation (UNESCO) University Twining Network (FIP UNESCO UNITWIN) in Africa. The survey URL link was further customised and distributed via social media and short message service platforms including Facebook®, Twitter®, WhatsApp® and Blackberry Messenger®. Respondents were encouraged to assist by forwarding the survey URL link to their colleagues and contacts. Email reminders were thereafter forwarded monthly through the aforementioned media until the end of the study. Due to the non-availability of reliable estimates on the number of pharmacists per country organisation represented in FIP, a sampling frame was not feasible and survey respondents were therefore recruited consecutively until the end of the study period.

### Survey questionnaire

An anonymous online questionnaire developed and validated in a previous study [26] was used. The questionnaire was fully reproduced from the GbCF v1 and comprised of 105 questions. Five questions related to demographic information while the remaining questions referred to the 100 GbCF v1 behavioural statements (labelled B1 through B100, and hereafter referred to as ‘behaviours’). These behaviours are grouped under the 20 competency domains and four broad competency clusters in the framework (Table 1).

**Table 1:** Components of the GbCF v1 framework

Cluster	Composition and description
Pharmaceutical public health	Four behaviours labelled B1–B4 grouped under two competencies: ‘health promotion’ and ‘medicines information and advice’
Pharmaceutical care	25 behaviours labelled B5–B29 grouped under six competencies: ‘assessment of medicines’, ‘dispensing’, ‘medicines’, ‘monitor medicines therapy’, ‘patient consultation and diagnosis’
Organisation and management	32 behaviours labelled B30–B61 grouped under six competencies: ‘budget and reimbursement’, ‘human resource management’, ‘improvement of service’, ‘procurement’, ‘supply chain and management’, and ‘work place management’
Professional and personal	39 behaviours labelled B62–B100 grouped under six competencies: ‘communication skills’, ‘continuing professional education’, ‘legal and regulatory practice’, ‘professional and ethical practice’, ‘quality assurance and research in the workplace’, ‘self-management’

### Data analysis

Survey data was collected electronically, without transformation and analysed using the Statistical Package for the Social Sciences (SPSS) version 22. To ensure data quality, a random 10% of the total survey sample was reviewed for coding errors with missing values replaced with code 999. Respondents’ perception of relevance to practice of each of the behaviours was evaluated using a 4-point Likert scale. Respondents were required to rank each of the 100 GbCF v1 behaviours as ‘not relevant’, of ‘low relevance’, ‘relevant’ or ‘highly relevant’ to their practice. For the purpose of analysis and to ensure the results produced could be meaningfully interpreted, the response categories in the Likert scale were further aggregated. The ‘highly relevant’ and ‘relevant’ ratings were condensed into one category: ‘relevant’, while the ‘low relevance’ and ‘not relevant’ ratings remained distinct categories. Agreement was evaluated by comparing the proportion (frequency and percentage) of the total ratings in the three categories. Consensus on relevance to practice was attained when not more than 10% of the respondents ranked a given behaviour as ‘not relevant’. This threshold was defined empirically based on previous research involving pharmacists from 64 countries [26].

Pearson’s chi-square (*χ*^2^) test was used to assess homogeneity in the survey sample. The test of homogeneity was undertaken to ascertain whether the sample could be treated as a group irrespective of the number of replies received per country. The *χ*^2^ test was also used to evaluate the relationship between weighting of relevance and area of practice for behaviours that showed a lack of consensus. In order to assess inter-country variability in responses, multivariate analysis of variance (MANOVA) via Pillia’s trace test (*V*) was used to evaluate weighting of relevance per country per competency with confirmatory post hoc analysis conducted using Bonferroni correction.

### Ethical consideration

Formal ethical approval from the research ethics committee was not required for this study as it did not involve the use of identifiable patient information or data, rather the study recruited pharmacists and sought their views by virtue of their professional roles. However, consent to participate was sought from the respondents prior to completing the survey questionnaire. Participation was voluntary, and confidentiality was maintained at all times with responses remaining anonymous. All data collected in the research were stored in an encrypted database with hard copies kept in locked filling cabinets at the Department of Practice and Policy, UCL School of Pharmacy, United Kingdom. Access to study data was restricted to the three researchers directly involved with the study.

## Results

### Demography

Four hundred and sixty-nine pharmacists from 14 countries in Africa responded to the survey. Over half of the survey respondents were female (54%). The mean length of practice was 7.7 years (SD ± 8.1 years; min–max 1–43 years). Respondents with less than 5 years practice experience made up 47% of the sample while pre-registration candidates/pharmacy students in their last year (internship) comprised 5.5%. The majority of the respondents were in hospital practice (56.7%). Table 2 shows the summary of the distribution of survey replies per country and area of pharmacy practice.

**Table 2:** Survey replies per country per area of pharmacy practice

Area of practice	Country *N* (%)				
	Kenya	Ghana	Nigeria	South Africa	Others ^a^
Academic pharmacy	1 (1.0)	4 (4.3)	11 (6.6)	21 (32.8)	6 (14.0)
Administrative pharmacy	6 (5.8)	13 (14.0)	4 (2.4)	2 (3.1)	4 (9.3)
Community pharmacy	2 (1.9)	10 (10.8)	41 (24.7)	10 (15.6)	10 (23.3)
Hospital pharmacy	89 (86.4)	60 (64.5)	80 (48.2)	23 (35.9)	14 (32.6)
Industrial pharmacy	1 (1.0)	3 (3.2)	26 (15.7)	6 (9.4)	5 (11.6)
Others^b^	4 (3.9)	3 (3.2)	4 (2.4)	2 (3.1)	4 (9.3)
Total sample	103 (22)	93 (19.8)	166 (35.4)	64 (13.6)	43 (9.2)

^a^Countries with fewer than 50 replies each [includes Zambia [15], Egypt [8], Zimbabwe [5], Uganda [3], Lesotho [3], Tunisia [5], Namibia, Ethiopia Sudan and Tanzania (1 each)]

^b^Areas of pharmacy practice with fewer than 20 replies [includes pharmacy information [11], military and emergency [2] and laboratory and medicines control pharmacy [4]]

### Sample homogeneity

Four countries—Kenya, Nigeria, South Africa and Ghana—each had more than 50 replies and made up 91% of the sample. Ten countries—Ethiopia, Egypt, Lesotho, Uganda, Tunisia, Namibia, Sudan, Tanzania, Zambia and Zimbabwe—had fewer than 20 survey replies each. The observed disparity in number of replies indicated two cluster groups: a ‘high response group’ made up of countries with more than 50 replies each, and a ‘low response group’ made up of countries with fewer than 20 replies each. The number of replies also varied for the four competency clusters in the framework. Table 3 shows a summary of the distribution of replies for each competency cluster per country group.

**Table 3:** Distribution of survey replies per competency cluster per country response group

Competency cluster	Behaviour label	Country response group (*N*)^a^	
		Countries with high response^b^	Countries with low response^c^
Pharmaceutical public health	B1–B4	426	43
Pharmaceutical care	B5–B29	350	36
Organisation and management	B30–B61	295	33
Professional and personal	B62–B100	273	32

^a^Countries with more than 50 replies each were regrouped as the high response group while countries with fewer than this were the low response group

^b^Includes Ghana, Kenya, Nigeria and South Africa

^c^Includes Zambia, Egypt, Zimbabwe, Uganda, Lesotho, Tunisia, Namibia, Ethiopia Sudan and Tanzania

**Fig. 1:**
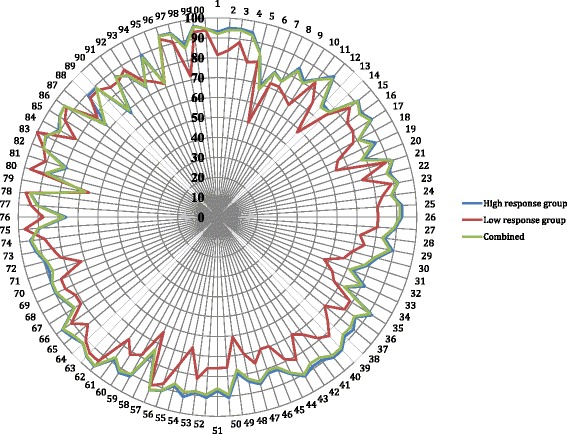
Percentage of total ratings in the ‘relevant’ category per behaviour (not inclusive of ratings in the ‘low relevance’ and ‘not relevant’ categories). Numbers on the circumference refer to behaviour labels (1 through 100) while numbers on the vertical axis refer to percentage of total ratings

The test for homogeneity in the survey sample showed that distribution of the ratings in the response categories (that is, in the ‘relevant’, ‘low relevance’ and ‘not relevant’ categories) was strongly associated with the country group for 11 behaviours [(*p* < 0.01); Table 4]. These 11 behaviours were distributed across the four competency clusters in no observable pattern. The outcome of the *χ*^2^ test implied homogeneity in responses between the country groups for 89% of the GbCF v1 behaviours. The low counts (frequency of less than 5) observed in the table cells within the group with low number of replies (*N* < 20 per country; Table 4) suggested an absence of data in this group rather than disparity in responses between countries. Given the *χ*^2^ test known property of being imprecise in ‘small’ samples [27–29], it is likely that the test overestimated the relationship between the responses and the country group. Based on this, sample homogeneity was assumed and the survey replies were subsequently analysed as a group. Figure 1 shows the percentage of ratings in the ‘relevant’ category for the low response, high response, and combined group of countries, respectively. The total ratings in the combined country groups indicate that at least 70% of survey respondents ranked all the behaviours in the framework as ‘relevant’. This was not inclusive of the ‘not relevant’ and ‘low relevant’ ratings.

**Table 4:** Behaviours showing association with country response group

Cluster	Competency	Behaviour	High response countries (*N*)			Low response countries (*N*)			*χ*^2^ value	*p*
			Not relevant	Low relevance	Relevant	Not relevant	Low relevance	Relevant		
Pharmaceutical public health	Health promotion	[B1] Assess primary healthcare needs	10	20	396	1*	7	35	9.67	*0.01*
	Medicines information and advice	[B4] Identify sources, retrieve, evaluate, assess and disseminate relevant medicines information according to patients needs	2*	22	402	1*	8	34	1.12	*0.01*
Pharmaceutical care	Assessment of medicines	[B6] Identify and act upon medicines interactions	40	68	242	3*	15	18	9.56	*0.01*
	Dispensing	[B12] Dispense devices (e.g. inhalers)	11	18	321	0*	9	27	20.54	*< 0.001*
	Medicines	[B19] Ensure appropriate medicine route, dose, time, form and response for individual patients	22	21	307	5	7	24	12.52	*0.002*
Organisation and management	Human resource management	[B37] Participate and collaborate, advice in therapeutic decision-making and use appropriate decision referral in a multi-disciplinary team	11	23	261	4*	6	23	9.4	*0.01*
	Improvement of service	[B42] Resolve, follow-up and prevent medicines related problems	10	23	262	1*	9	23	12.8	*< 0.001*
	Procurement	[B44] Develop and implement a contingency plan for shortages	11	27	257	5	2*	26	8.5	*0.01*
	Supply chain management	[B54] Implement system for documentation and record keeping	11	13	271	3*	8	22	22.35	*0.001*
	Work place management	[B61] Recognise and manage pharmacy resources (e.g. financial, infrastructure)	9	26	260	5	3*	25	10.71	*0.01*
Professional and personal	Quality assurance and research in the workplace	[B94] Implement, conduct and maintain a report system of pharmacovigilance (e.g. report adverse drug reactions)	16	11	246	2*	6	24	11.88	*0.003*

*Counts of less than 5

### Overall perception of relevance

Consensus on relevance to practice (*N* ‘not relevant’ < 10%) was obtained for 90 behaviours in the questionnaire. This included all the behaviours in the ‘pharmaceutical public health’ cluster, 84% in the ‘pharmaceutical care’, 90% in ‘organisation and management’ and 92% in the ‘professional and personal’ cluster, respectively (please see Tables 5, 6, 7 and 8). The 10 behaviours with more than 10% of the total ratings in the ‘not relevant’ category, suggested disagreement on relevance to practice (Table 9). This observed disagreement was associated with area of pharmacy practice for six of the ‘disagreed’ behaviours (Table 9; *P* < 0.05).

**Table 9:** Distribution of the ‘not relevant’ ratings in relation to area of pharmacy practice for the 10 behaviours showing disagreement

Cluster	Competencies	Behaviours	Area of practice (%*N* ‘not relevant’)						*χ*^2^ value	*p*
			Acad.	Admin.	Comm.	Hosp.	Indus.	Others^a^		
Pharmaceutical care	Assessment of medicines	[B6] Identify, prioritise and act upon medicine-medicine interactions; medicine-disease interactions; medicine-patient interactions; medicines-food interactions (*n* = 43)	20	*9.5*	0	11.6	21.9	7.1	23.5	*0.01*
	Dispensing	[B13] Document and act upon dispensing errors (*n* = 50)	14.3	19	20	*7.6*	21.9	35.7	36.9	*< 0.001*
		[B14] Implement and maintain a dispensing error report system and a ‘near misses’ report system (*n* = 43)	17.1	14.3	20.3	*7.1*	12.5	14.3	15.9	0.1
	Medicines	[B20] Package medicines to optimise safety [ensuring appropriate re-packaging and labelling of the medicines] (*n* = 39)	20	*9.5*	16.9	*4.4*	25	14.3	31.9	*< 0.001*
Organisation and management	Budget and reimbursement	[B32] Ensure appropriate claim for reimbursement (*n* = 40)	24	*5*	10.9	*8.8*	24	27.3	20.1	0.03
		[B34] Ensure proper reference sources for service reimbursement (*n* = 34)	18.2	*5*	10.9	*7.8*	16	27.3	16.8	0.08
	Workplace management	[B58] Ensure production schedules are appropriately planned and managed (*n* = 35)	15.2	*5*	19.6	*6.7*	20	18.2	17.4	0.08
Professional and personal	Quality assurance and research in the workplace	[B87] Apply research findings and understand the benefit risk [e.g. pre-clinical, clinical trials, experimental clinical-pharmacological research and risk management] (*n* = 34)	12.5	*5*	20.9	10.2	9.1	0	20.4	0.03
		[B90] Ensure appropriate quality control tests are performed and managed appropriately (*n* = 39)	12.5	*0*	25.6	11.9	9.1	9.1	22.8	*0.01*
		[B95] Initiate and implement audit and research activities (*n* = 33)	12.5	*5*	20.9	*9*	9.1	0	15.78	0.11

*Acad.* academic pharmacy, *Admin.* administrative pharmacy, *Comm.* community pharmacy, *Hosp.* hospital pharmacy, *Indus.* industrial pharmacy

^a^Includes areas of pharmacy practice with fewer than 20 replies (these were pharmacy information [11], military and emergency pharmacy [2] and laboratory and medicines control pharmacy [4])

The disagreement was also associated with ‘patient-facing role’ in practice area [(*P* < 0.05); Table 10]. A higher percentage of the respondents in the ‘non-patient’ facing areas of practice (this means area of pharmacy practice with little or no daily patient interactions such as industrial and academic pharmacy) ranked the behaviours that showed disagreement in the ‘pharmaceutical care’ (B6, B13, B14, B20) and ‘organisation and management’ cluster (B32, B34, B58) as ‘not relevant’ compared to respondents in the ‘patient-facing’ practice areas (such as hospital and community pharmacy) (Table 10). The converse was true for the research-related (B87 and B95) and quality control (B90) behaviours (Table 10).

**Table 10:** Distribution of the ‘not relevant’ ratings in relation to patient-facing component in area of pharmacy practice for the 10 behaviours showing disagreement

Cluster	Competency	Behaviour	Patient-facing sectors* (*N*%)			Non patient-facing sectors^¶^ (*N*%)			*χ*^2^ value	*p*
			Not relevant	Low relevance	Relevant	Not relevant	Low relevance	Relevant		
Pharmaceutical care	Assessment of medicines	[B6] Identify, prioritise and act upon medicine-medicine interactions; medicine-disease interactions; medicine-patient interactions; medicine-food interactions (*n* = 43)	9.1	21.1	69.8	18.2	22.7	59.1	6.3	0.04
	Dispensing	[B13] Document and act upon dispensing errors (*n* = 50)	11.4	11.4	77.2	18.2	11.4	70.5	2.8	0.25
		[B14] Implement and maintain a dispensing error report system and a ‘near misses’ report system (*n* = 43)	10.1	13.4	76.5	14.8	6.8	78.4	3.87	0.15
	Medicines	[B20] Package medicines to optimise safety [ensuring appropriate re-packaging and labelling of the medicines] (*n* = 39)	7.4	11.7	80.9	19.3	13.6	67	11.45	0.003
Organisation and management	Budget and reimbursement	[B32] Ensure appropriate claim for the reimbursement (*n* = 40)	10	18.4	71.6	19.2	10.3	70.5	6.56	0.04
		[B34] Ensure proper reference sources for service reimbursement (*n* = 34)	9.2	16.8	74	14.1	17.9	67.9	1.72	0.44
	Work place management	[58] Ensure the production schedules are appropriately plan and manage (*n* = 35)	9.6	18.4	72	14.1	7.7	78.2	5.74	0.06
Professional and personal	Quality assurance and research in the workplace	[B87] Apply research findings and understand the benefit risk [e.g. pre-clinical, clinical trials, experimental clinical-pharmacological research and risk management] (*n* = 34)	11.7	18.2	82.4	9.5	8.1	70.1	5.02	0.08
		[B90] Ensure appropriate quality control tests are performed and managed appropriately (*n* = 39)	14.3	21.2	64.5	8.1	6.8	85.1	11.72	0.003
		[B95] Initiate and implement audit and research activities (*n* = 33)	11.3	19.9	68.8	9.5	8.1	82.4	6.19	0.05

*Pharmacy areas involving daily patient interactions including hospital and community pharmacy

^¶^Pharmacy areas not involving daily patient interactions including industrial, academic, administrative, laboratory and medicine control, and pharmacy information

**Table 5:** Overall rating of behaviours within the pharmaceutical public health cluster

Pharmaceutical public health competencies (*n* = 469)	Not relevant		Low relevance		Relevant		Group
	Count	Row (*N*%)	Count	Row (*N*%)	Count	Row (*N*%)	
Health promotion							
B1. HP | Assess the primary healthcare needs (taking into account the cultural and social setting of the patient)	11	2.30	27	5.8	431	91.9	1
B2. HP | Advise on health promotion, disease prevention and control, and healthy lifestyle	2	0.4	26	5.5	441	94	1
Medicines information and advice							
B3. MIA | Counsel patients on the appropriate use of medicines and devices (including the selection, use, contraindications, storage, and side effects of non-prescription and prescription medicines) taking into account patients preferences	3	0.60	23	4.90	443	94.4	1
B4. MIA | Identify sources, retrieve, evaluate, organise, assess and disseminate relevant medicines information according to the needs of patients and clients and provide appropriate information	3	0.60	30	6.40	436	92.9	1

Behaviours that showed agreement (this means, *N* ‘not relevant’ ≤ 10%) were categorised as group 1; group 2 behaviours showed disagreement on relevance (*N* ‘not relevant’ > 10%)

**Table 6:** Overall rating of behaviours in the pharmaceutical care cluster

Pharmaceutical care competencies (*n* = 386)	Not relevant		Low relevance		Relevant		Group
	Count	Row (*N*%)	Count	Row (*N*%)	Count	Row (*N*%)	
Assessment of medicines							
B5. AM | Appropriately select medicines (e.g. according to the patient, hospital, government policy, etc.)	18	4.7	40	10.4	328	85	1
B6. AM | Identify, prioritise and act upon medicine-medicine interactions; medicine-disease interactions; medicine-patient interactions; medicines-food interactions	43	11.1	83	21.5	260	67.4	2
Compounding medicines							
B7. CM | Prepare pharmaceutical medicines (e.g. extemporaneous, cytotoxic medicines), determine the requirements for preparation (calculations, appropriate formulation, procedures, raw materials, equipment etc.)	25	6.5	61	15.8	300	77.7	1
B8. CM | Compound under the good manufacturing practice for pharmaceutical (GMP) medicines	35	9.1	61	15.8	290	75.1	1
Dispensing							
B9. D | Accurately dispense medicines for prescribed and/or minor ailments and monitor the dispense (re-checking the medicines)	19	4.9	43	11.2	324	83.9	1
B10. D | Accurately report defective or substandard medicines to the appropriate authorities	26	6.7	53	13.7	307	79.5	1
B11. D | Appropriately validate prescriptions, ensuring that prescriptions are correctly interpreted and legal	16	4.1	41	10.6	329	85.2	1
B12. D | Dispense devices (e.g. Inhaler or a blood glucose meter)	11	2.8	27	7	348	90.2	1
B13. D | Document and act upon dispensing errors	50	13	44	11.4	292	75.6	2
B14. D | Implement and maintain a dispensing error report system and a ‘near misses’ report system	43	11.1	46	11.9	297	76.9	2
B15. D | Label the medicines (with the required and appropriate information)	14	5.2	20	3.6	352	91.2	1
B16. D | Learn from and act upon previous ‘near misses’ and ‘dispensing errors	19	4.9	27	7	340	88.1	1
Medicines							
B17. M | Advise patients on proper storage conditions of the medicines and ensure that medicines are stored appropriately (e.g. humidity, temperature, expiry date, etc.)	17	4.9	19	4.4	350	90.7	1
B18. M | Appropriately select medicines formulation and concentration for minor ailments (e.g. diarrhoea, constipation, cough, hay fever, insect bites, etc.)	33	8.5	42	10.9	311	80.6	1
B19. M | Ensure appropriate medicines, route, time, dose, documentation, action, form and response for individual patients	27	7	28	7.3	331	85.8	1
B20. M | Package medicines to optimise safety (ensuring appropriate re-packaging and labelling of the medicines)	39	10.1	47	12.2	300	77.7	2
Monitor medicines therapy							
B21. MMT | Apply guidelines, medicines formulary system, protocols and treatment pathways	11	4.9	19	2.9	356	92.2	1
B22. MMT | Ensure therapeutic medicines monitoring, impact and outcomes (including objective and subjective measures)	22	5.7	26	6.7	338	87.6	1
B23. MMT | Identify, prioritise and resolve medicines management problems (including errors)	12	3.1	19	4.9	355	92	1
Patient consultation and diagnosis							
B24. PCD | Apply first aid and act upon arranging follow-up care	18	4.7	29	7.5	339	87.8	1
B25. PCD | Appropriately refer	14	3.6	18	4.7	354	91.7	1
B26. PCD | l Assess and diagnose based on objective and subjective measures	12	3.1	21	5.4	353	91.5	1
B27. PCD | Discuss and agree with the patients the appropriate use of medicines, taking into account patients preferences	14	3.6	30	7.8	342	88.6	1
B28. PCD | Document any intervention (e.g. document allergies, medicines and food, in patient medicines history)	21	5.4	43	11.1	322	83.4	1
B29. PCD | Obtain, reconcile, review, maintain and update relevant patient medication and diseases history	18	4.7	28	7.3	340	88.1	1

Group: 1 = agreement, 2 = disagreement

**Table 7:** Overall rating of behaviours in the organisation and management cluster

Organisation and management competencies (*n* = 328)	Not relevant		Low relevance		Relevant		Group
	Count	Row (*N*%)	Count	Row (*N*%)	Count	Row (*N*%)	
Budget and reimbursement							
B30. BR | Acknowledge the organisational structure	16	4.9	45	13.7	267	81.4	1
B31. BR | Effectively set and apply budgets	20	6.1	53	16.2	255	77.7	1
B32. BR | Ensure appropriate claim for the reimbursement	40	12.2	54	16.5	234	71.4	2
B33. BR | Ensure financial transparency	21	6.4	45	13.7	262	79.9	1
B34. BR | Ensure proper reference sources for service reimbursement	34	10.4	56	17.1	238	72.5	2
Human resources management							
B35. HRM | Demonstrate organisational and management skills (e.g. Know, understand and lead on medicines management; risk management; self-management; time management; people management; project management; policy management.)	8	2.4	23	7	297	90.6	1
B36. HRM | Identity and manage human resources and staffing issues	13	4	37	11.3	278	84.8	1
B37. HRM | Participate, collaborate, advice in therapeutic decision-making and use appropriate referral in a multi-disciplinary team	15	4.6	29	8.8	284	86.6	1
B38. HRM | Recognise and manage the potential of each member of the staff and utilise systems for performance management (e.g. carry out staff appraisals)	12	3.7	33	10.1	283	86.3	1
B39. HRM | Recognise the value of the pharmacy team and of a multidisciplinary team	7	2.1	25	7.6	296	90.2	1
B40. HRM | Support and facilitate staff training and professional development	9	2.7	23	7	296	90.2	1
Improvement of service							
B41. IS | Identify and implement new services (according to local needs)	7	2.1	35	10.7	286	87.2	1
B42. IS | Resolve, follow up and prevent medicines related problems	11	3.4	32	9.8	285	86.9	1
Procurement							
B43. P | Access reliable information and ensure the most cost-effective medicines in the right quantities with the appropriate quality	12	3.7	20	6.1	296	90.2	1
B44. P | Develop and implement contingency plan for shortages	16	4.9	29	8.8	283	86.3	1
B45. P | Efficiently link procurement to formulary, to push/pull system (supply chain management) and payment mechanisms	18	5.5	44	13.4	266	81.1	1
B46. P | Ensure there is no conflict of interest	19	5.8	43	13.1	266	81.1	1
B47. P | Select reliable suppliers of high-quality products (including appropriate selection process, cost effectiveness, timely delivery)	16	4.9	32	9.8	280	85.4	1
B48. P | Supervise procurement activities	23	7.0	34	10.4	271	82.6	1
B49. P | Understand the tendering methods and evaluation of tender bids	25	7.6	50	15.2	253	77.1	1
Supply chain and management							
B50. SCM | Demonstrate knowledge in store medicines to minimise errors and maximise accuracy	15	4.6	20	6.1	293	89.3	1
B51. SCM | Ensure accurately verification of rolling stocks	16	4.9	30	9.1	282	86	1
B52. SCM | Ensure effective stock management and running of service with the dispensary	17	5.5	18	5.2	293	89.3	1
B53. SCM | Ensure logistics of delivery and storage	16	4.9	23	7	289	88.1	1
B54. SCM | Implement a system for documentation and record keeping	14	4.3	21	6.4	293	89.3	1
B55. SCM | Take responsibility for quantification of forecasting	22	7.0	23	6.7	283	86.3	1
Work place management							
B56. WPM | Address and manage day to day management issues	8	2.4	20	6.1	300	91.5	1
B57. WPM | Demonstrate the ability to take accurate and timely decisions and make appropriate judgments	8	2.4	15	4.6	305	93	1
B58. WPM | Ensure the production schedules are appropriately plan and manage	35	10.7	52	15.9	241	73.5	2
B59. WPM | Ensure the work time is appropriately plan and manage	10	3	23	7	295	89.9	1
B60. WPM | Improve and manage the provision of pharmaceutical services	8	2.4	20	6.1	300	91.5	1
B61. WPM | Recognise and manage pharmacy resources (e.g. financial, infrastructure)	14	4.3	29	8.8	285	86.9	1

Group: 1 = agreement, 2 = disagreement

**Table 8:** Overall rating of behaviours in the professional and personal cluster

Professional and personal competencies (*n* = 305)	Not relevant		Low relevance		Relevant		Group
	Count	Row (*N*%)	Count	Row (*N*%)	Count	Row (*N*%)	
Communication skills							
B62. CS | Communicate clearly, precisely and appropriately while being a mentor or tutor	4	1.3	6	2	295	96.7	1
B63. CS | Communicate effectively with health and social care staff, support staff, patients, carer, family relatives and clients/customers, using lay terms and checking understanding	4	1.3	9	3	292	95.7	1
B64. CS | Demonstrate cultural awareness and sensitivity	6	2.0	17	5.6	282	92.5	1
B65. CS | Tailor communications to patient needs	5	1.6	15	4.9	285	93.4	1
B66. CS | Use appropriate communication skills to build, report and engage with patients, health and social care staff and voluntary services (e.g. verbal and non-verbal)	3	< 0.1	10	3.3	292	95.7	1
Continuing professional development (CPD)							
B67. CPD | Document CPD activities	10	3.3	36	11.8	259	84.9	1
B68. CPD | Engage with students/interns/residents	12	3.9	28	9.2	265	86.9	1
B69. CPD | Evaluate currency of knowledge and skills	8	2.6	25	8.2	272	89.2	1
B70. CPD | Evaluate learning	8	2.6	25	8.2	272	89.2	1
B71. CPD | Identify if expertise needed outside the scope of knowledge	9	3.0	31	10.2	265	86.9	1
B72. CPD | Identify learning needs	7	2.3	28	9.2	270	88.5	1
B73. CPD | Recognise own limitations and act upon them	5	1.6	20	6.6	280	91.8	1
B74. CPD | Reflect on performance	4	1.3	14	4.6	287	94.1	1
Legal and regulatory practice							
B75. LRP | Apply and understands regulatory affairs and the key aspects of pharmaceutical registration and legislation	7	2.3	23	7.5	275	90.2	1
B76. LRP | Apply knowledge in relation to the principals of business economics and intellectual property rights including the basics of patent interpretation	16	5.2	54	17.7	235	77.1	1
B77. LRP | Be aware of and identify the new medicines coming to the market	4	1.3	25	8.2	276	90.5	1
B78. LRP | Comply with legislation for drugs with the potential for abuse	5	1.6	11	3.6	289	94.8	1
B79. LRP | Demonstrate knowledge in Marketing and Sale	24	7.9	75	24.6	206	67.5	1
B80. LRP | Engage with health and medicines policies	3	< 0.1	31	10.2	271	88.9	1
B81. LRP | Understand the steps needed to bring a medicinal product to the market including the safety, quality, efficacy and pharmacoeconomic assessments of the product	18	5.9	41	13.4	246	80.7	1
Professional and ethical practice							
B82. PEP | Demonstrate awareness of local/national codes of ethics	5	1.6	14	4.6	286	93.8	1
B83. PEP | Ensure confidentiality (with the patient and other healthcare professionals)	4	1.3	10	3.3	291	95.4	1
B84. PEP | Obtain patient consent (it can be implicit in occasions)	10	3.3	21	6.9	274	89.8	1
B85. PEP | Recognise own limitations	5	1.6	14	4.6	286	93.8	1
B86. PEP | Take responsibility for own action and for patient care	5	1.6	11	3.6	289	94.8	1
Quality assurance and research in the work place							
B87. QARWP | Apply research findings and understand the benefit risk (e.g. pre-clinical, clinical trials, experimental clinical-pharmacological research and risk management)	34	11.1	48	15.7	223	73.1	2
B88. QARWP | Audit quality of service (ensure that they meet local and national standards and specifications)	26	8.5	34	11.1	245	80.3	1
B89. QARWP | Developed and implement Standing Operating Procedures (SOP’s)	17	5.6	17	5.6	271	88.9	1
B90. QARWP | Ensure appropriate quality control tests are performed and managed appropriately	39	12.8	54	17.7	212	69.5	2
B91. QARWP | Ensure medicines are not counterfeit and quality standards	16	5.2	24	7.9	265	86.9	1
B92. QARWP | Identify and evaluate evidence-base to improve the use of medicines and services	16	5.2	32	10.5	257	84.3	1
B93. QARWP | Identify, investigate, conduct, supervise and support research at the workplace (enquiry-driven practice)	29	9.5	48	15.7	228	74.8	1
B94. QARWP | Implement, conduct and maintain a report system of pharmacovigilance (e.g. report adverse drug reactions)	17	5.9	18	5.6	270	88.5	1
B95. QARWP | Initiate and implement audit and research activities	33	10.8	52	17.5	220	72.1	2
Self-management							
B96. SM | Apply assertiveness skills (inspire confidence)	5	1.6	9	3	291	95.4	1
B97. SM | Demonstrate leadership and practice management skills, initiative and efficiency	4	1.3	12	3.9	289	94.8	1
B98. SM | Document risk management (e.g. critical incidents)	14	4.6	28	9.2	263	86.2	1
B99. SM | Ensure punctuality	1	< 0.1	10	3.3	294	96.4	1
B100. SM | Prioritise work and implement innovative ideas	4	1.3	12	3.9	289	94.8	1

Group: 1 = agreement, 2 = disagreement

### Perception of relevance per competency per country

Inter-country variability in responses was assessed for each of the 20 competencies in the questionnaire. For ease of interpretation, the analysis included 426 replies from four countries: Kenya, Nigeria, South Africa and Ghana with the 10 countries that had fewer than 20 survey replies each excluded. The result showed similarity in weighting of relevance for the competencies in the ‘pharmaceutical public health’ (Pillia’s trace *V* = 0.025, *F* = 1.809, df = 6, *p* = 0.094), and ‘professional and personal’ (Pillia’s trace *V* = 0.084, *F* = 1.270, df = 18, *p* = 0.2) clusters, respectively. Specific inter-country variability was observed in the ‘pharmaceutical care’ (Pillia’s trace *V* = 0.083, *F* = 1.624, df = 18, *p* = 0.045), and ‘organisation and management’ (Pillia’s trace *V* = 0.136, *F* = 2.279, df = 18, *p* = 0.002) clusters.

Confirmatory post hoc analysis using Bonferroni correction showed the inter-country variability in responses observed within the ‘pharmaceutical care’ cluster was in three behaviours: B6 [‘assessment of medicine (AM)]’ and B25 and B27 [‘patient consultation and diagnosis (PCD)’] competencies. This was between South Africa and Ghana [in B6: *N* ‘relevant’ = 47% vs 71%)], Nigeria and Kenya [in B25: *N* ‘relevant’ = 98 vs 89%], and Nigeria and Ghana [in B27: *N* ‘relevant’ = 98 vs 85%)]. In spite of the observed disparity in weighting of relevance, only South Africa showed a lack of consensus on relevance (*N* ‘not relevant’ = 15%) for this cluster and this was in the B6 behaviour.

The disparity in the organisation and management cluster was in B32 [‘budget and reimbursement (BR)’], B45 [‘procurement (P)’] and B55 [‘supply chain and management (SCM)’] competencies. More of the respondents from Ghana rated the B32, B45 and B55 behaviours ‘relevant’ compared to Nigeria (75%, 92%, 94% vs 70%, 70%, 79%, respectively). A lack of consensus on relevance was observed in the South Africa and Nigeria group for the B32 (*N* ‘not relevant’ = 11%) and B49 (*N* ‘not relevant’ = 11%) behaviours, respectively. Since only Nigeria and South Africa showed a lack of consensus (*N* ‘not relevant’ > 10%) on relevance to practice for three behaviours in the framework, it suggests that the inter-country variability in weighting of relevance observed in this study indicated differences in perception of ‘degree of relevance’ between countries. This variability is likely due to the disparity in sample composition in the respective countries given that Kenya had a higher percentage of the respondents in hospital practice (86%) while Nigeria and South Africa on the other hand had less than 50% respectively. Also, more than a third of the respondents from South Africa were in academic pharmacy compared to Nigeria, Kenya and Ghana with less than 7% each.

## Discussion

The disagreement observed in 10 behaviours in the GbCF v1 framework was mainly related to respondents’ area of pharmacy practice and the corresponding patient-facing involvement, a finding that is consistent with evidence from previous research [26]. The disagreement in the four behaviours under the ‘pharmaceutical care’ cluster observed in academic and industrial pharmacy is also in line with the scope of practice of pharmacists in these areas given that they are not routinely involved in activities related to medicine assessment and medicines use. This also explains the disagreement observed in the three behaviours under the ‘organisation and management’ cluster.

On the other hand, the disagreement in the research-related behaviours (B87 and B95) under the ‘professional and personal’ cluster was not fully explained by area of pharmacy practice or ‘patient-facing’ involvement. A high percentage (*N* > 10%) of the respondents in academic and community pharmacy rated these same behaviours ‘not relevant’, thereby adding to the increasing body of evidence from other studies that suggest that pharmacists are not routinely involved in research [30–33] and perceive their research-related roles to be of low importance [34–37]. It also corroborates the findings of published studies from Australia [38], United Kingdom [39] and Thailand [40] that show that pharmacists generally perceive research-related behaviours and competencies included in developmental frameworks to be relatively low in relevance and rank them accordingly. Time constraints due to workload and a lack of supporting environment for pharmacy research are some of the barriers to participation in research-related activities in the workplace reported in existing literature [38, 41, 42]. Given that the survey respondents in our study included international pharmacists from different areas of pharmacy practice and with varying length of practice experience, this finding highlights the need to scale up efforts to build research capacity in this region.

Of particular interest is the finding that a high percentage (*N* > 15%) of community pharmacists from the countries represented in this survey ranked two dispensing-related behaviours: B13 (document and act upon dispensing errors) and B14 (implement and maintain a dispensing error report system and a near miss report system) ‘not relevant’ to practice. This suggests community pharmacy respondents from these countries do not routinely carry out these activities although this may have been due to the response rate and/or that the pharmacists were self-selecting. However, available evidence suggests this may also be related to the peculiarities of community pharmacy practice in countries with severe health workforce shortages such as Nigeria [43] and Zambia [44]. Studies show that dispensing activities in community pharmacies in Nigeria are mainly carried out by pharmacy assistants and in some instances, by sales personnel or clerks [43].

Furthermore, the finding may also be related to evidence that suggest that many countries including those in Africa either lack a defined medication error reporting system [45, 46] or where available such systems are primarily independent and/or based within a specific healthcare facility [45, 47]. Given the broad similarities in pharmacy practice reported in countries within the African region [48] and published reports of high incidence of patient harm due to medication errors in some of the countries represented in this survey [49, 50]. This finding underscores the need to review current practice and incorporate robust dispensing and medication error reporting processes in community practice with oversight functions by pharmacists in order to assure patient safety.

Homogeneity in sample responses and the overall survey results indicate minimal disparity between countries in perception of relevance to practice for majority of the behaviours in the GbCF v1. This finding corroborates evidence from previous research [26] and provides evidence that was previously lacking on the relevance of the GbCF v1 competencies in these countries. The finding is in consonance with similar evidence from the field of medicine that demonstrates the relevance of the Canadian CanMEDS Physician Competency Framework to medical practice in the Netherlands [51], Denmark [52, 53] and Australia [54]. It is also in line with evidence from studies that show consensus between countries in Europe on the relevance of a core set of competencies for pharmacy education and practice [42, 55].

Although evidence from global studies have shown that continuous professional development (CPD) is mandatory in many countries in Africa, none of the countries represented in this survey have reported the availability of a validated competency framework for early career pharmacy practice [7, 56]. Studies conducted in Ghana, Ethiopia and Sudan suggest that the lack of a structured post-registration pathway for skills development contribute to the comparatively lower levels of job satisfaction shown by early career pharmacists in these countries [57–59]. Our findings therefore provide preliminary evidence on the validity of a core set of competencies that can be further adapted to country context and used to design skill development and learning activities for pre- and in-service early career pharmacy practitioners in these countries. Our findings also suggests the feasibility of adapting the GbCF v1 to develop country-specific frameworks for use in facilitating performance improvement and identifying learning needs particularly in the four countries with comparatively high number of replies in this study.

This study has some limitations. The length of the survey questionnaire (105 questions presented over six pages) may have negatively impacted on the number of replies received. Findings from a meta-analysis of randomised controlled trials show that the odds of a response decreases by more than half as the number of pages of a survey questionnaire increases [OR 0.39, 95% CI 0.34 to 0.45] [60]. This was particularly obvious with the consistent decrease in number of replies per additional competency cluster in the survey questionnaire (Additional file 1). Nonetheless, research also demonstrates that the variation in response rates per page of a questionnaire does not affect the quality of the overall responses received [61].

Online surveys are generally associated with low response rates particularly because it restricts the target populations to individuals with internet access [62, 63]. This implies that potential respondents without Internet access were excluded from this survey, a feature that is significant given that our study was conducted in countries that have been shown to have comparatively higher cost and lower Internet accessibility [64, 65]. However, the geographical location of the survey population, limited resources and time available for this research precluded the use of a telephone or postal survey. Participant self-selection and the non-probabilistic sample obtained via the purposive and snowball sampling technique in our study likely limits the generalisability of our findings. Studies show that self-selected participants are likely to be more intrinsically motivated than the general population [66]. This non-random sampling method was undertaken due to challenges with obtaining a sampling frame for the respective countries. For this same reason, it was difficult to estimate an optimal sample size a priori and to calculate a response rate. In spite of these limitations, the methods used in our study are established and pragmatic evidence-based approaches in pharmacy practice research [26, 38–40]. Furthermore, given that our study was an exploratory survey, our findings provide useful information on pharmacists’ perception of relevance to practice of the competencies and behaviours contained in the GbCF v1 framework in the countries represented.

Further work is necessary to qualitatively explore expert opinion and obtain insight from other stakeholders including policy-makers and pharmacy leaders on the validity of these competencies in the respective countries. Also, a larger scale validation study is needed to obtain further inputs from practitioners in non-patient-facing roles such as industrial, administrative and academic pharmacy in this region. This will provide an opportunity for further review of the GbCF v1 competencies in relation to these specific areas of practice.

## Conclusion

The majority (90%) of the competencies in the framework as relevant to practice for the respondents in this survey, although there are some emergent differences in weighting of relevance between the countries represented. Overall, the findings provide preliminary evidence that was previously lacking on the relevance of the GbCF v1 competencies to pharmacy practice in these countries.

## Additional file

Additional file 1: Survey Questionnaire. (DOCX 63 kb)

## Appendix

**Table 11:** FIP member organisations and the respective countries in Africa

S/N	Organisation	Country
1.	Pharmacy Council of Ghana	Ghana
2.	Pharmaceutical Society of Kenya	Kenya
3.	Ordre National des Pharmaciens de Madagascar	Madagascar
4.	Pharmaceutical Association of Mauritius	Mauritius
5.	Conseil Regional des Pharmaciens d’officine du Nord	Morocco
6.	Conseil National de l’ordre des Pharmaciens du Mali	Mali
7.	Pharmaceutical Society of Nigeria	Nigeria
8.	Rwanda Pharmacists Association	Rwanda
9.	Ordre National des Pharmaciens du Senegal	Senegal
10.	Pharmaceutical Society of South Africa	South Africa
11.	Sudanese Pharmacists Union	Sudan
12.	Pharmaceutical society of Uganda	Uganda
13.	Pharmaceutical Society of Zambia	Zambia
14.	Pharmaceutical Society of Zimbabwe	Zimbabwe
15.	Ordre National des Pharmaciens du Burkina Faso	Burkina Faso
16.	Conseil National de L’ordre des Pharmaciens du Cameroun	Cameroun
17.	Ordre National des Pharmaciens du Tchad N’Djamena	Chad
18.	Comseil National de l’ordre des Pharmaciens du Congo	Congo
19.	Pharmaceutical Society of Egypt	Egypt
20.	Syndicate of Pharmacists in Arab Republic of Egypt	Egypt
21.	Eritean Pharmaceutical Association	Eritea
22.	Ethiopian Pharmaceutical Association	Ethiopia
23.	Conseil National de l’ordre des Pharmaciens de Guinea Conakry	Republic of Guinea
24.	Conseil National de l’ordre des Pharmaciens de Cote d’Ivoire	Cote d’Ivoire

## Abbreviations

AM: Assessment of medicine competency
BR: Budget and reimbursement competency
D: Dispensing competency
FIP: The International Pharmaceutical Federation
FIPEd: The International Pharmaceutical Federation Education Initiative
GbCF v1: Global Competency Framework version 1
M: Medicines competency
OR: Odds ratio
P: Procurement competency
PCD: Patient consultation and diagnosis competency
SCM: Supply chain and management competency
SPSS v22: Statistical Package for the Social Sciences version 22
