# Supplementary material for: A survey of pharmacists’ perception of foundation level competencies in African countries
Source: Hum Resour Health. 2018 Apr 2;16:16. doi: 10.1186/s12960-018-0280-1 (PMC5879617; doi:10.1186/s12960-018-0280-1)
Supplement: Supplementary file 1 — Survey Questionnaire. (DOCX 63 kb) [file 12960_2018_280_MOESM1_ESM.docx]

Survey Questionnaire

**Development Team**

**Competency Development Domain | Validation of the GbCF v1 in an African Context**

**Global Competency Framework v1**

Competent pharmacists have the potential to improve therapeutic outcomes and patients’ quality of life. Before competence can be determined, the specific competencies and behaviours that contribute to it must be identified.

The aim of the overall project is to develop a global competency framework (GbCF) that includes behavioural statements, which can be adaptable to local needs and cultural contexts relevant to a global pharmaceutical workforce. This would be consistent with other health professions (eg, physicians) who have achieved a harmonised global consensus about competency.

In the initial phase of the project, documents/frameworks were collected from around the world. Through consultation and review, these have been synthesised into this draft global version. Now we need your feedback about relevance or how well these behavioural statements fit your practice.

As a pharmacist, your contribution is highly valuable, ensuring that the core competencies and behaviours you feel important to your daily work are incorporated in the global competency framework draft version survey. I hereby invite you to share your thoughts.

**Instructions:**

We will now ask you some specific questions about behavioural statements that constitute the provision of pharmaceutical services. The items are divided into four separate clusters: pharmaceutical public health, pharmaceutical care, organisation and management, and professional/personal competencies.

Please think only about your own pharmaceutical practice and then rate each individual behavioural statement as relevant or not relevant to your practice.

Please follow the directions and answer all questions. This is an anonymous, confidential questionnaire; responses will be reported in aggregate and no one respondent will be identifiable. Please answer honestly, reflecting actual practice rather than desired practice.

This questionnaire should take no more than 20 minutes of your time. All contributions are highly valued, and we appreciate your time and effort.

**Many thanks for your contribution.**

For further information or if you have any enquiries about this questionnaire please contact [Andreia Bruno](mailto:andreiafbruno@gmail.com) or [Arit Udoh](mailto:arith_udoh@yahoo.co.uk).

**Demographic Information:**

| **Country of residence:** | ____________________ |
| --- | --- |
| **Years Qualified:** | _______ |
| **Gender:** | Female |
|  | Male |
| **Current Status:** | Internship (still pre-registration while at university/college) |
|  | Pre-registration candidate (not yet registered or licensed) |
|  | Licensed/registered pharmacist |
| **Area of Practice:** | Academic Pharmacy |
|  | Administrative Pharmacy |
|  | Clinical Biology |
|  | Community Pharmacy |
|  | Hospital Pharmacy |
|  | Industrial Pharmacy |
|  | Laboratories and Medicine Control Section |
|  | Military and Emergency Pharmacy |
|  | Pharmacy Information |
|  | Other: |

**Please think only about your own pharmaceutical practice and then rate each individual behavioural statement as relevant or not relevant to your practice.**

| **1. Pharmaceutical Public Health Competencies** | | | | |  |  |  |  |
| --- | --- | --- | --- | --- | --- | --- | --- | --- |
| **Competencies** | | | **Behaviours** | | **Highly Relevant** | **Relevant** | **Low Relevant** | **Not Relevant** |
|  |  | | |  | | | | |
| 1.1 Health promotion | | | 1.1.1 Assess the primary healthcare needs (taking into account the cultural and social setting of the patient) | |  |  |  |  |
|  |  |  | 1.1.2 Advise on health promotion, disease prevention and control, and healthy lifestyle | |  |  |  |  |
|  |  | | |  | | | | |
| 1.2 Medicines information and advice | | | 1.2.1 Counsel population on the safe and rational use of medicines and devices (including the selection, use, contraindications, storage, and side effects of non-prescription and prescription medicines) | |  |  |  |  |
|  |  |  | 1.2.2 Identify sources, retrieve, evaluate, organise, assess and disseminate relevant medicines information according to the needs of patients and clients and provide appropriate information | |  |  |  |  |
|  |  | | |  | | | | |
| **2. Pharmaceutical Care Competencies** | | | | |  |  |  |  |
| **Competencies** | | | **Behaviours** | | **Highly Relevant** | **Relevant** | **Low Relevant** | **Not Relevant** |
|  |  | | |  | | | | |
| 2.1 Assessment of medicines | | | 2.1.1 Appropriately select medicines (e.g. according to the patient, hospital, government policy, etc) | |  |  |  |  |
|  |  |  | 2.1.2 Identify, prioritise and act upon medicine-medicine interactions; medicine-disease interactions; medicine-patient interactions; medicines-food interactions | |  |  |  |  |
|  |  | | |  | | | | |
| 2.2 Compounding medicines | | | 2.2.1 Prepare pharmaceutical medicines (e.g. extemporaneous, cytotoxic medicines), determine the requirements for preparation (calculations, appropriate formulation, procedures, raw materials, equipment etc.) | |  |  |  |  |
|  |  |  | 2.2.2 Compound under the good manufacturing practice for pharmaceutical (GMP) medicines | |  |  |  |  |
|  |  | | |  | | | | |
| 2.3 Dispensing | | | 2.3.1 Accurately dispense medicines for prescribed and/or minor ailments and monitor the dispense (re-checking the medicines) | |  |  |  |  |
|  |  |  | 2.3.2 Accurately report defective or substandard medicines to the appropriate authorities | |  |  |  |  |
|  |  |  | 2.3.3 Appropriately validate prescriptions, ensuring that prescriptions are correctly interpreted and legal | |  |  |  |  |
|  |  |  | 2.3.4 Dispense devices (e.g. Inhaler or a blood glucose meter) | |  |  |  |  |
|  |  |  | 2.3.5 Document and act upon dispensing errors | |  |  |  |  |
|  |  |  | 2.3.6 Implement and maintain a dispensing error reporting system and a ‘near misses’ reporting system | |  |  |  |  |
|  |  |  | 2.3.7 Label the medicines (with the required and appropriate information) | |  |  |  |  |
|  |  |  | 2.3.8 Learn from and act upon previous ‘near misses’ and ‘dispensing errors’ | |  |  |  |  |
|  |  | | |  | | | | |
| 2.4 Medicines | | | 2.4.1 Advise patients on proper storage conditions of the medicines and ensure that medicines are stored appropriately (e.g. humidity, temperature, expiry date, etc.) | |  |  |  |  |
|  |  |  | 2.4.2 Appropriately select medicines formulation and concentration for minor ailments (e.g. diarrhoea, constipation, cough, hay fever, insect bites, etc.) | |  |  |  |  |
|  |  |  | 2.4.3 Ensure appropriate medicines, route, time, dose, documentation, action, form and response for individual patients | |  |  |  |  |
|  |  |  | 2.4.4 Package medicines to optimise safety (ensuring appropriate re-packaging and labelling of the medicines) | |  |  |  |  |
|  |  | | |  | | | | |
| 2.5 Monitor medicines therapy | | | 2.5.1 Apply guidelines, medicines formulary system, protocols and treatment pathways | |  |  |  |  |
|  |  |  | 2.5.2 Ensure therapeutic medicines monitoring, impact and outcomes (including objective and subjective measures) | |  |  |  |  |
|  |  |  | 2.5.3 Identify, prioritise and resolve medicines management problems (including errors) | |  |  |  |  |
|  |  | | |  | | | | |
| 2.6 Patient consultation and diagnosis | | | 2.6.1 Apply first aid and act upon arranging follow-up care | |  |  |  |  |
|  |  |  | 2.6.2 Appropriately refer | |  |  |  |  |
|  |  |  | 2.6.3 Assess and diagnose based on objective and subjective measures | |  |  |  |  |
|  |  |  | 2.6.4 Discuss and agree with the patients the appropriate use of medicines, taking into account patients’ preferences | |  |  |  |  |
|  |  |  | 2.6.5 Document any intervention (e.g. document allergies, medicines and food, in patient medicines history) | |  |  |  |  |
|  |  |  | 2.6.6 Obtain, reconcile, review, maintain and update relevant patient medication and diseases history | |  |  |  |  |
|  |  | | |  | | | | |
| **3. Organisation and Management Competencies** | | | | |  |  |  |  |
| **Competencies** | | | **Behaviours** | | **Highly Relevant** | **Relevant** | **Low Relevant** | **Not Relevant** |
|  |  | | |  | | | | |
| 3.1 Budget and reimbursement | | | 3.1.1 Acknowledge the organisational structure | |  |  |  |  |
|  |  |  | 3.1.2 Effectively set and apply budgets | |  |  |  |  |
|  |  |  | 3.1.3 Ensure appropriate claim for the reimbursement | |  |  |  |  |
|  |  |  | 3.1.4 Ensure financial transparency | |  |  |  |  |
|  |  |  | 3.1.5 Ensure proper reference sources for service reimbursement | |  |  |  |  |
|  |  | | |  | | | | |
| 3.2 Human Resources management | | | 3.2.1 Demonstrate organisational and management skills (e.g. know, understand and lead on medicines management; risk management; self management; time management; people management; project management; policy management.) | |  |  |  |  |
|  |  |  | 3.2.2 Identity and manage human resources and staffing issues | |  |  |  |  |
|  |  |  | 3.2.3 Participate, collaborate, advise in therapeutic decision-making and use appropriate referral in a multi-disciplinary team | |  |  |  |  |
|  |  |  | 3.2.4 Recognise and manage the potential of each member of the staff and utilise systems for performance management (e.g. carry out staff appraisals) | |  |  |  |  |
|  |  |  | 3.2.5 Recognise the value of the pharmacy team and of a multidisciplinary team | |  |  |  |  |
|  |  |  | 3.2.6 Support and facilitate staff training and continuing professional development | |  |  |  |  |
|  |  | | |  | | | | |
| 3.3 Improvement of service | | | 3.3.1 Identify and implement new services (according to local needs) | |  |  |  |  |
|  |  |  | 3.3.2 Resolve, follow up and prevent medicines related problems | |  |  |  |  |
|  |  | | |  | | | | |
| 3.4 Procurement | | | 3.4.1 Access reliable information and ensure the most cost-effective medicines in the right quantities with the appropriate quality | |  |  |  |  |
|  |  |  | 3.4.2 Develop and implement contingency plan for shortages | |  |  |  |  |
|  |  |  | 3.4.3 Efficiently link procurement to formulary, to push/pull system (supply chain management) and payment mechanisms | |  |  |  |  |
|  |  |  | 3.4.4 Ensure there is no conflict of interest | |  |  |  |  |
|  |  |  | 3.4.5 Select reliable supplies of high-quality products (including appropriate selection process, cost effectiveness, timely delivery) | |  |  |  |  |
|  |  |  | 3.4.6 Supervise procurement activities | |  |  |  |  |
|  |  |  | 3.4.7 Understand the tendering methods and evaluation of tender bids | |  |  |  |  |
|  |  | | |  | | | | |
| 3.5 Supply chain and management | | 3.5.1 Demonstrate knowledge in store medicines to minimise errors and maximise accuracy | | |  |  |  |  |
|  |  | 3.5.2 Ensure accurate verification of rolling stocks | | |  |  |  |  |
|  |  | 3.5.3 Ensure effective stock management and running of service with the dispensary | | |  |  |  |  |
|  |  | 3.5.4 Ensure logistics of delivery and storage | | |  |  |  |  |
|  |  | 3.5.5 Implement a system for documentation and record keeping | | |  |  |  |  |
|  |  | 3.5.6 Take responsibility for quantification of forecasting | | |  |  |  |  |
|  |  | | |  | | | | |
| 3.6 Work place management | | | 3.6.1 Address and manage day to day management issues | |  |  |  |  |
|  |  |  | 3.6.2 Demonstrate the ability to take accurate and timely decisions and make appropriate judgments | |  |  |  |  |
|  |  |  | 3.6.3 Ensure the production schedules are appropriately planned and managed | |  |  |  |  |
|  |  |  | 3.6.4 Ensure the work time is appropriately planned and managed | |  |  |  |  |
|  |  |  | 3.6.5 Improve and manage the provision of pharmaceutical services | |  |  |  |  |
|  |  |  | 3.6.6 Recognise and manage pharmacy resources (e.g. financial, infrastructure) | |  |  |  |  |

| **4. Professional/Personal Competencies** | | | |  |  |  |  |
| --- | --- | --- | --- | --- | --- | --- | --- |
| **Competencies** | | **Behaviours** | | **Highly Relevant** | **Relevant** | **Low Relevant** | **Not Relevant** |
|  |  | |  | | | | |
| 4.1 Communication skills | | 4.1.1 Communicate clearly, precisely and appropriately while being a mentor or tutor | |  |  |  |  |
|  |  | 4.1.2 Communicate effectively with health and social care staff, support staff, patients, carer, family relatives and clients/customers, using lay terms and checking understanding | |  |  |  |  |
|  |  | 4.1.3 Demonstrate cultural awareness and sensitivity | |  |  |  |  |
|  |  | 4.1.4 Tailor communications to patient needs | |  |  |  |  |
|  |  | 4.1.5 Use appropriate communication skills to build, report and engage with patients, health and social care staff and voluntary services (e.g. verbal and non-verbal) | |  |  |  |  |
|  |  | |  | | | | |
| 4.2 Continuing Professional Development (CPD) | | 4.2.1 Document CPD activities | |  |  |  |  |
|  |  | 4.2.2 Engage with students/interns/residents | |  |  |  |  |
|  |  | 4.2.3 Evaluate currency of knowledge and skills | |  |  |  |  |
|  |  | 4.2.4 Evaluate learning | |  |  |  |  |
|  |  | 4.2.5 Identify if expertise needed outside the scope of knowledge | |  |  |  |  |
|  |  | 4.2.6 Identify learning needs | |  |  |  |  |
|  |  | 4.2.7 Recognise own limitations and act upon them | |  |  |  |  |
|  |  | 4.2.8 Reflect on performance | |  |  |  |  |
|  |  | |  | | | | |
| 4.3 Legal and regulatory practice | | 4.3.1 Apply and understand regulatory affairs and the key aspects of pharmaceutical registration and legislation | |  |  |  |  |
|  |  | 4.3.2 Apply knowledge in relation to the principals of business economics and intellectual property rights including the basics of patent interpretatio | |  |  |  |  |
|  |  | 4.3.3 Be aware of and identify the new medicines coming to the market | |  |  |  |  |
|  |  | 4.3.4 Comply with legislation for drugs with the potential for abuse | |  |  |  |  |
|  |  | 4.3.5 Demonstrate knowledge in marketing and sales | |  |  |  |  |
|  |  | 4.3.6 Engage with health and medicines policies | |  |  |  |  |
|  |  | 4.3.7 Understand the steps needed to bring a medicinal product to the market including the safety, quality, efficacy and pharmacoeconomic assessments of the product | |  |  |  |  |
|  |  | |  | | | | |
| 4.4 Professional and ethical practice | | 4.4.1 Demonstrate awareness of local/national codes of ethics | |  |  |  |  |
|  |  | 4.4.2 Ensure confidentiality (with the patient and other healthcare professionals) | |  |  |  |  |
|  |  | 4.4.3 Obtain patient consent (it can be implicit on occasion) | |  |  |  |  |
|  |  | 4.4.4 Recognise own professional limitations | |  |  |  |  |
|  |  | 4.4.5 Take responsibility for own action and for patient care | |  |  |  |  |
|  |  | |  | | | | |
| 4.5 Quality Assurance and Research in the work place | | 4.5.1 Apply research findings and understand the benefit risk (e.g. pre-clinical, clinical trials, experimental clinical-pharmacological research and risk management) | |  |  |  |  |
|  |  | 4.5.2 Audit quality of service (ensure that they meet local and national standards and specifications) | |  |  |  |  |
|  |  | 4.5.3 Develop and implement Standing Operating Procedures (SOP’s) | |  |  |  |  |
|  |  | 4.5.4 Ensure appropriate quality control tests are performed and managed appropriately | |  |  |  |  |
|  |  | 4.5.5 Ensures medicines are not counterfeit and quality standards | |  |  |  |  |
|  |  | 4.5.6 Identify and evaluate evidence-base to improve the use of medicines and services | |  |  |  |  |
|  |  | 4.5.7 Identify, investigate, conduct, supervise and support research at the workplace (enquiry-driven practice) | |  |  |  |  |
|  |  | 4.5.8 Implement, conduct and maintain a reporting system of pharmacovigilance (e.g. report Adverse Drug Reactions) | |  |  |  |  |
|  |  | 4.5.9 Initiate and implement audit and research activities | |  |  |  |  |
|  |  | |  | | | | |
| 4.6 Self-management | | 4.6.1 Apply assertiveness skills (inspire confidence) | |  |  |  |  |
|  |  | 4.6.2 Demonstrate leadership and practice management skills, initiative and efficiency | |  |  |  |  |
|  |  | 4.6.3 Document risk management (e.g. critical incidents) | |  |  |  |  |
|  |  | 4.6.4 Ensure punctuality | |  |  |  |  |
|  |  | 4.6.5 Prioritise work and implement innovative ideas | |  |  |  |  |
